# An Electronic-Nose Sensor Node Based on a Polymer-Coated Surface Acoustic Wave Array for Wireless Sensor Network Applications

**DOI:** 10.3390/s110504609

**Published:** 2011-04-28

**Authors:** Kea-Tiong Tang, Cheng-Han Li, Shih-Wen Chiu

**Affiliations:** Department of Electrical Engineering, National Tsing Hua University, No. 101, Sec. 2, Kuang-Fu Road, Hsinchu 30013, Taiwan; E-Mails: chli@larc.ee.nthu.edu.tw (C.-H.L.); swchiu@larc.ee.nthu.edu.tw (S.-W.C.)

**Keywords:** electronic nose, wireless sensor network, sensor node, SAW sensor array

## Abstract

This study developed an electronic-nose sensor node based on a polymer-coated surface acoustic wave (SAW) sensor array. The sensor node comprised an SAW sensor array, a frequency readout circuit, and an Octopus II wireless module. The sensor array was fabricated on a large *K*^2^ 128° YX LiNbO3 sensing substrate. On the surface of this substrate, an interdigital transducer (IDT) was produced with a Cr/Au film as its metallic structure. A mixed-mode frequency readout application specific integrated circuit (ASIC) was fabricated using a TSMC 0.18 μm process. The ASIC output was connected to a wireless module to transmit sensor data to a base station for data storage and analysis. This sensor node is applicable for wireless sensor network (WSN) applications.

## 1. Introduction

Wireless sensor networks (WSNs) [1,2] comprise a number of light-weight low-power sensor nodes. Each node is equipped with a number of sensors, interface electronics, and a wireless communication module to deliver the collected data. Applications of WSNs have been proposed in areas as diverse as health care [3], monitoring of industrial equipment [4], precision horticulture [5], volcanology [6], habitat observation [7] and monitoring of engines [8]. Issues associated with air pollution have been attracting increased attention because air quality has a direct influence on human health. Combining gas sensors within a WSN is an effective means to monitor urban air pollution [9]. However, it has proven unfeasible for general applications because each gas sensor has a corresponding target gas, requiring the installation of numerous sensors to monitor multiple gases. Electronic nose (E-Nose) systems have shown great potential in odor detection, analysis, and recognition. E-Noses first appeared in 1987, and were designed to mimic mammalian olfactory systems [10]. Olfactory processes do not perceive odors using only a single receptor, but rather detect and recognize scents according to an array of multiple receptors, with each combination representing a different odor. The permutations and combinations enable the recognition of complex gasses such as volatile organic compounds (VOCs) [11,12] and the odor of fruits [13]. Incorporating a WSN with an E-Nose to form a sensor array would greatly expand the scope of odor classification and practical applicability.

A polymer-coated surface acoustic wave (SAW) array is one of the best choices to achieve high sensitivity in applications requiring the detection of organic gasses [14,15]. Currently SAW devices are being used for a variety of chemical applications because of their high sensitivity, fully reversible behavior, and high signal-to-noise ratio [16]. For example, piezoelectric substrates are used to transform energy from mechanical strain to electric signals. Interdigital transducers (IDTs) are input and output comb-like metal electrodes, used as energy transformation structures on the surface of selected substrates. When an AC voltage is applied to an input IDT, dynamic strain is induced, launching a wave across the surface of the substrate. The induced surface wave propagates through the active sensing region, to be received and transformed into electrical signals by the output IDT. A change in mass on the active sensing region can be detected, according to changes in magnitude and phase shift in the AC signal between the input and output IDTs. To increase the selectivity and sensitivity of the sensor, a variety of polymers are used to coat the active sensing region of the SAW sensors to absorb molecules of the target gas. The traditional approach to building up a SAW-based E-Nose system has been to connect the SAW array to a spectrum analyzer or a frequency counter to monitor frequency shifts in the SAW devices. Unfortunately, neither of these options is practical for portable or WSN applications, due to their bulk and high price. To resolve this problem, a small, inexpensive frequency readout scheme is required.

Fabricating frequency readout circuitry using integrated circuits (IC) not only reduces the size of the system, but also the cost of mass production [17]. In recent years, designers have had the option to integrate general WSN platforms [18,19] with sensor devices for a variety of applications. In this study, we report an efficient E-Nose sensor node comprising a 2 × 2 non-continuous chemical SAW sensor array chip using MEMS technology self-assembled with polymer coatings on the active sensing region [20,21], application specific integrated circuit (ASIC) chip for a mixed signal interface, and a WSN platform (Octopus II). The low-power, high-resolution ASIC chip was fabricated using a TSMC 0.18 μm 1P6M standard CMOS process. The ASIC chip was connected directly to an array of four SAW sensors, outputting frequency data representative of the sensor response. The output data was transmitted to a base station using an Octopus II wireless module. Results of measuring the chip and experimental data are presented in the following sections.

In Section 2, we introduce the proposed E-Nose gas sensor node and the experimental setup. In Section 3, we show the experimental results of the SAW sensor, the ASIC interface, and the fabricated sensor node. A brief conclusion is provided in Section 4.

## 2. Proposed E-Nose Sensor Node

**Figure 1.**
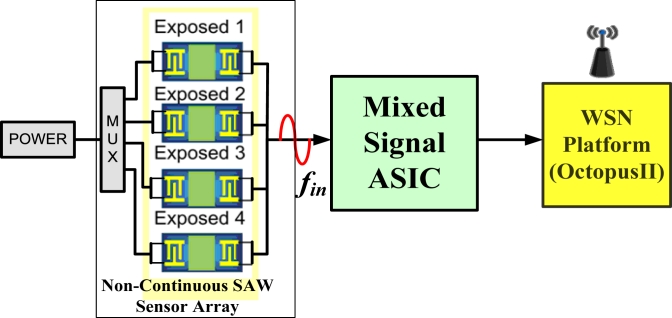
Block diagram of SAW sensor node.

Figure 1 presents a block diagram of the proposed E-Nose sensor node. The Sensor node comprises three major parts: an SAW sensor array, a mixed signal ASIC chip, and a WSN platform (Octopus II).

### 2.1. Surface Acoustic Wave (SAW) Sensor

In the late 1970s, various gas sensors were developed based on a variety of operational principles. Compared with other gas sensors, SAW-based gas sensors provide a high degree of sensitivity, reproducibility, and stability [22]. The sensing mechanism is based on the fact that volatile organic compounds are adsorbed on the surface of a substrate and an increase in mass loading causes a shift in frequency. The mechanism behind SAW sensors involves the input of voltage inducing an electric field between the IDTs. The piezoelectric effect induces dynamic strain on the substrate to launch the SAW, propagating across the surface of the substrate. At the receiving end, the IDT converts the mechanical signals to electric signals. Finally, fluctuations in the output frequency are measured. According to the design parameters from the simulation data of delta function model and the cross field model shown in Table 1, LiNbO_3_-based. SAW devices with IDTs of Cr/Au (20 nm/100 nm) were fabricated in a photolithography process with a center frequency of 117.4 MHz. The size of the SAW sensors was 3 mm × 2 mm.

### 2.2. SAW Sensor Array

**Figure 3.**
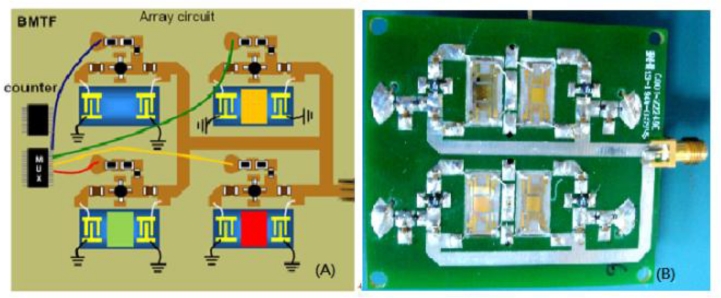
**(A)** The schematic array circuit and **(B)** The photo of a SAW array with 2 × 2 non-continuous working oscillators and SAW chips.

A SAW array was employed to fabricate a gas sensor with the advantages of small size, low cost, high sensitivity, and rapid response. As shown in Figure 3, the 2 × 2 non-continuous working oscillators were controlled by a multiplexing technique as a switching element [23].

**Figure 4.**
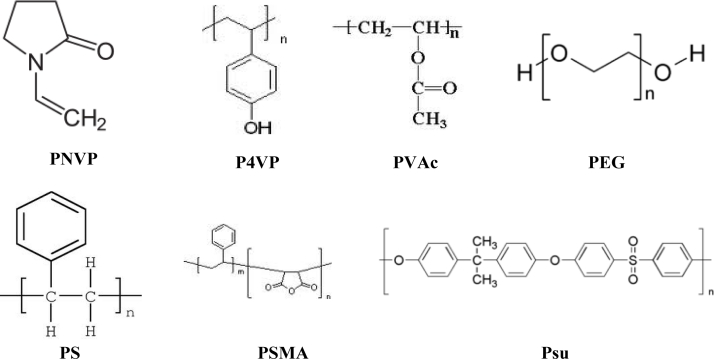
Seven polymer formulas selected as the sensitive films.

The film of the sensor operated like a smart skin, responsible for generating chemical signals from interactions between molecules and the film [24]. The sensing polymer film acted as the mucosa of the nasal cavity, playing an important role in the detection of gases. To enhance sensitivity and selectivity, seven polymers were selected as materials to be used in the sensitive film, spin-coated on the surface of the resonators of the array (Figure 4). These polymers included poly-*N*-vinylpyrrolidone (PNVP), poly-4-vinylphenol (P4VP), polyvinyl acetate (PVAc), polyethylene-glycol (PEG), polystyrene (PS), polystyrene-co-maleic anhydride (PSMA) and polysulfone (Psu).

In the E-Nose system, a sensor array was used to form patterns for use in the recognition of gases. In this study, the sensor array comprised four SAW sensor devices. These four sensors did not operate at the same time by controlling the oscillators non-continuously to minimize power consumption and interference from crosstalk [23]. In addition, by operating the SAW array non-continuously, all sensor outputs could be connected, thereby only one interface readout circuit was needed, saving chip area and total cost. In this study, the non-continuous SAW sensor reached a stable output within 0.2 s after being switched on. Therefore, each sensor was switched on for 1s to ensure stable sensor output.

### 2.3. Mixed Signal Interface ASIC

There are many kinds of circuit implementations to detect SAW frequencies and/or phases. One way is to convert the SAW frequency into a voltage signal. This approach wastes a large portion of the voltage range because the shift in the frequency of the sensor does not exceed a certain amount [25]. Another way measures phase differences, but the resolution is only at the level of MHz [26]. In this study, the center frequency was at 117.4 MHz, and the maximum frequency shift did not exceed 1 MHz, representing as little less than 1% that of the center frequency. Therefore, if a direct frequency to voltage converter was adopted, assuming a linear conversion from frequency to voltage, approximately 99% of the voltage range was wasted, making it difficult to improve resolution.

**Figure 5.**
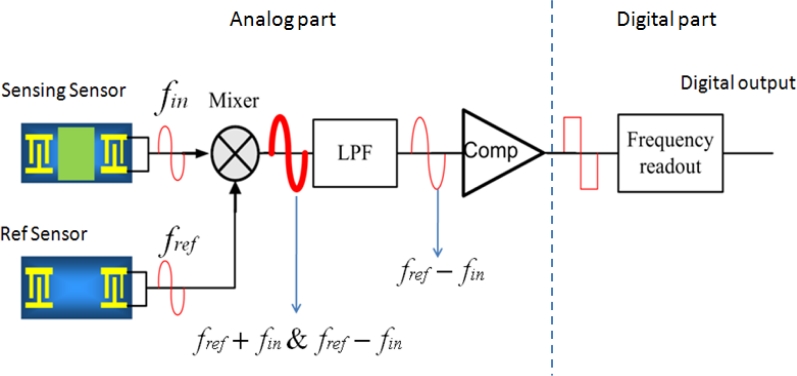
Block diagram of the mixed-signal interface chip.

In this study, we implemented a mixed-signal interface chip, as shown in Figure 5. The first stage of the chip was analog, including a mixer, low-pass filter, and comparator. The function of the analog stage was to modulate, filter, and convert the signal from the sensor into a square wave. The second stage of the chip was a digital readout to detect changes in sensor frequency. To read out the sensor signal (frequency change), the frequency of the sensor *f_in_* was subtracted from that of a reference sensor *f_ref_*. This resulted in a decrease in the frequency change *f_ref_*–*f_in_* compared to *f_ref_*. Because the amplitude of the signal requiring conversion was smaller, resolution could be improved and power consumption could be reduced. The reference sensor was not coated with a polymer membrane; therefore, the variations in its frequency due to input gas were very small. Nevertheless, the reference sensor was still influenced by the same environment parameters as the sensing sensor, such as temperature and humidity. Subtracting *f_in_* from *f_ref_* helped to eliminate the background effect of the sensor.

We integrated a mixer into the frontend of the analog section, the output of which had a high frequency term *f_ref_* + *f_in_* and a low frequency term *f_ref_*–*f_in_*. After the mixer, a low-pass filter passed the low frequency term *f_ref_*–*f_in_*, to a comparator to generate a square wave output to the digital stage.

**Figure 6.**
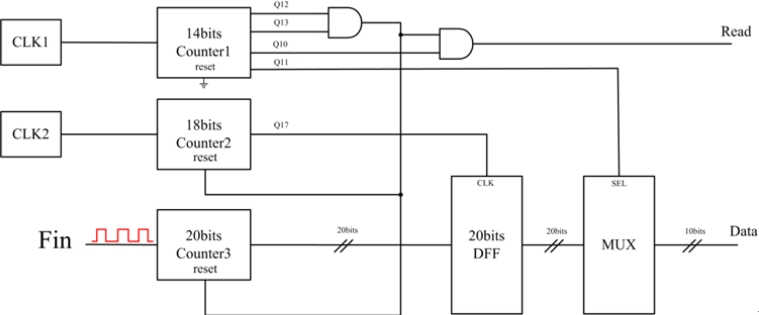
Schematic of digital frequency readout circuit.

A digital frequency readout circuit received the output from the comparator and reported the frequency data. The main idea was to use three counters with D flip-flops (DFF) for storage, as shown in Figure 6.

Counter 2 provided a fixed sampling time T_S_ = 2^17^ × T_CLK2_, where T_CLK2_ was the clock period of CLK2. The input signal (Fin) went into Counter 3 as the clock. When Counter 3 counted for the time 2^17^ × T_CLK2_, the MSB of Counter 2 triggered the DFF and stored the output data (*D*) of Counter 3 at this moment. Counter 1 generated control signals for the multiplexor (MUX), the reset signal of Counter 3, and the Read signal for the Octopus II. The multiplexor (MUX) output the data stored in the DFF. The input signal (Fin) could be calculated later by:

(1) $${\text{F}}_{\text{in}}=\frac{\text{D}}{2^{17}}\times {\text{f}}_{\text{CLK}2}=\frac{\text{D}}{{\text{T}}_{\text{S}}}$$

According to Equation (1), the resolution of the mixed-signal interface chip depends on the sampling time T_S_. Theoretically, a finer resolution can be achieved by a longer sampling time. For example, a sample time of one second corresponds to a resolution of 1 Hz (each bit in Counter 3 represents 1 Hz), and a sampling time of 100 ms corresponds to a resolution of 10 Hz (each bit in Counter 3 represents 10 Hz). Since each sensor was switched on for 1 s, a sampling time of 1 s would be too long. Moreover, 10 Hz resolution was enough for detection. Consequently, a sampling time of 100 ms was chosen in this work.

### 2.4. WSN Platform

The Octopus II [27,28] WSN platform was selected to transmit sensing data. Because the output of the frequency readout circuitry was not a fast-changing signal, it could be connected directly to the Octopus 50-pin extension connector. Octopus II includes MSP430F1611, a USB Interface, Inverted F, and SMA Type Antenna. The basic features of Octopus II are as follows: RF range is approximately 450 m, board size is 80 mm × 31 mm, maximum output power is approximately 10 dBm, compatible with IEEE 802.15.4 (ZigBee), and operates using 2 × AA batteries (3.3 V 2,700 mAh). Detailed information can be obtained from the website (http://www.wsnc.ntu.edu.tw/Files/Octopus--_0913_V1_2%20[----].pdf).

### 2.5. Gas Experimental Setup

The sensor data was transmitted through a wireless module to a personal computer operating as a base station for data storage, processing, and analysis. To verify the E-Nose sensor node, three tests had to be performed: (1) a gas experiment to validate the SAW sensor array; (2) a configuration test to verify the accuracy of the mixed-signal readout chip; (3) a verification test to determine the correctness of the wireless transmission.

**Figure 7.**
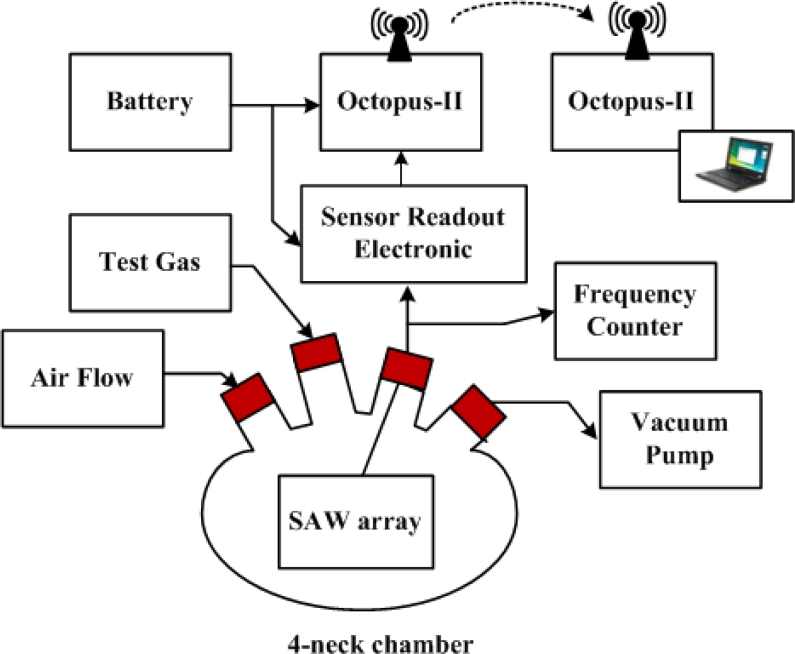
Gas experimental setup.

Figure 7 illustrates the gas experimental setup, in which seven different sensing membranes were used in the SAW sensor array. The array was tested using ethanol and acetone, and the complete system was tested in a 1L 4-neck bottle chamber. The four windows were for the vacuum pump, test gas input, wire connection, and air flow valve. Considering that the membrane may have absorbed excessive water vapors, we first baked the sensor array for 20 minutes at 90 °C, prior to measurement, to reduce the water vapor interference from inside the membrane. One cycle of gas testing involved four steps:

1. Pumping: The system was operated stably under vacuum pumping for 5 minutes.
2. Injection: Following the stabilization of frequency, the vacuum pump was turned off and the air flow valve was closed. Test gas was injected into the chamber, and the device responded for 5 minutes.
3. Air: The vacuum pump was turned on and the air flow valve was opened to clean the chamber for 5 minutes.
4. The air flow valve closed, returning to step (1).

The total time required for one cycle was approximately 15 minutes. The system responded very rapidly to the test gas causing a frequency shift within 3 minutes. To test the accuracy of the mixed-signal readout chip, the output was compared with a frequency counter.

## 3. Experimental Results and Discussion

### 3.1. SAW Gas Sensor

**Figure 8.**
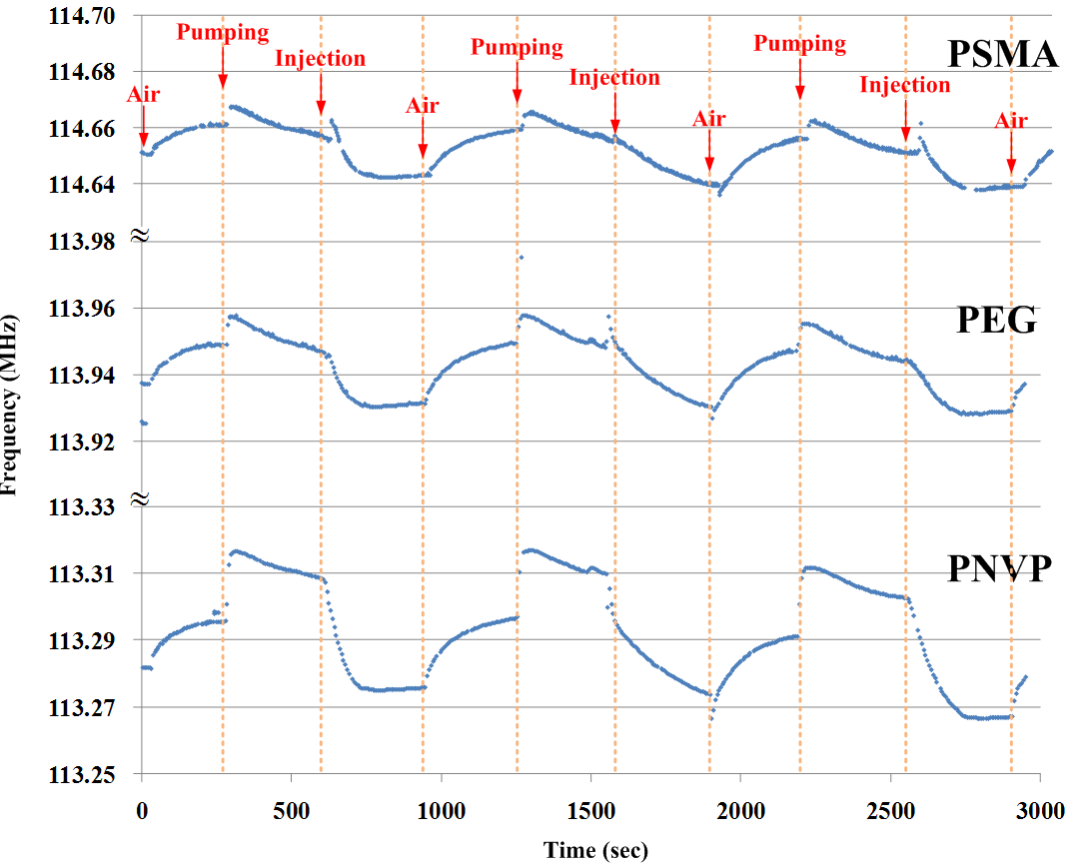
Typical sensor response (gas: ethanol, membrane: PNVP).

The SAW array was tested using ethanol and acetone. Figure 8 shows a typical response of the sensors (coated with PMSA, PEG, and PNVP) exposed to ethanol. The experiment was repeated three times. The baseline seemed to drift due to temperature variation. The SAW devices are known to be sensitive to its environment parameters, especially temperature [29]. Currently, many SAW devices are manufactured with materials such as lithium niobate or lithium tantalite. The advantage of using these two materials is their high *K^2^* values, at the price of their high temperature coefficients. Our way to compensate the temperature effect is to use a reference SAW device without membrane.

### 3.2. Sensor Interface Measurement Results

To verify the accuracy of the interface chip, a signal generator was used to emulate the SAW signals. A spectrum analyzer measured the outputs of the mixer and the low-pass filter. The two input signals to the mixer were 117 MHz and 118 MHz sinusoidal wave with amplitude 0.5 V. As a result, the spectrum analyzer showed that the mixer output spectrum had two frequency components, 1 MHz and 235 MHz, and the low-pass filter output spectrum was 1 MHz. These results verified the function of the mixer and the low-pass filter.

**Figure 9.**
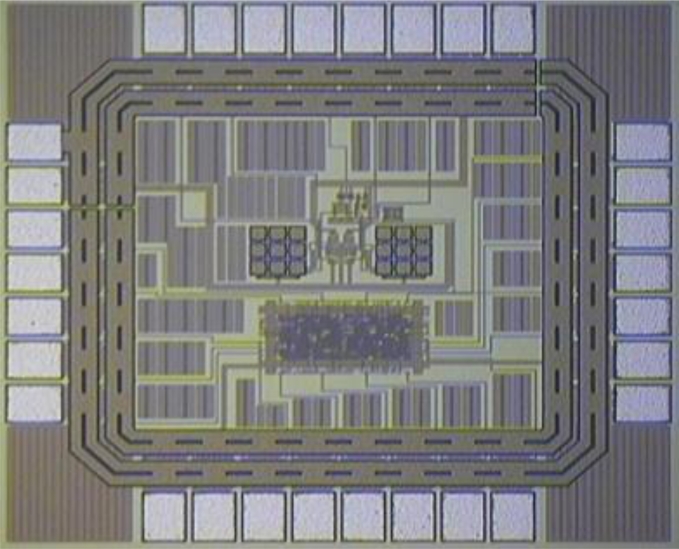
Die photo of this low-power mixed-signal SAW interface ASIC.

The output of the low-pass filter was sent to a comparator to convert the signal into a square wave, which was passed to the digital frequency readout circuit. To test the frequency readout circuit, a square wave was input to the circuit and into a frequency counter at the same time. The test frequencies were 10, 100, 1 k, 10 k, 100 k, and 1 MHz. Each frequency was sampled 1,000 times. The total test time was 100 seconds. In all the test results, the frequency readout circuit outputs were the same as the frequency counter. The power consumption for the entire chip was 1.48 mW using a power supply of 3.3 V. Figure 9 shows a photo of the die used in this low-power mixed-signal SAW interface ASIC. Table 2 provides a benchmark with other studies of the SAW interface circuit [25,26].

### 3.3. Sensor Node

**Figure 10.**
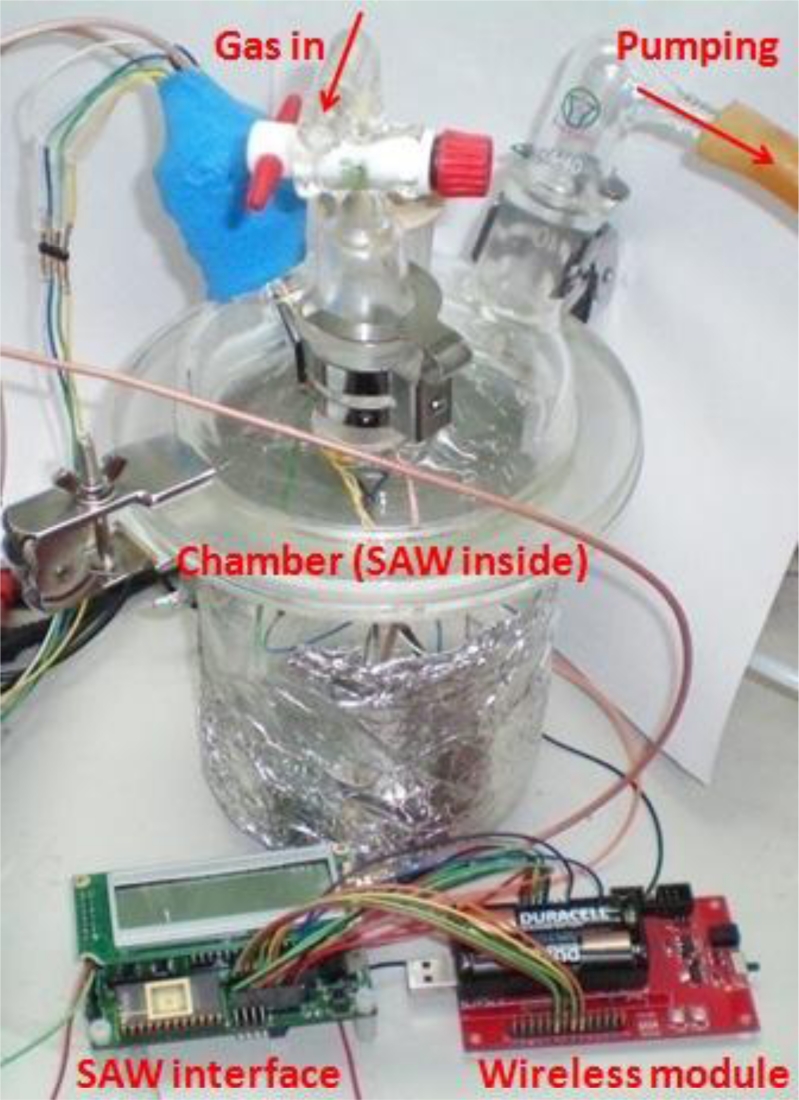
Test setup of the sensor node.

Figure 10 shows the test setup of the sensor node. The SAW array was connected to the interface chip, and the output was connected to the wireless module. Both the interface chip and the wireless module were running on two 1.5 V batteries. The wireless module transmitted the sensor data to a base station PC. This data was compared with the frequency counter output according to Equation (2):

(2) $$(\sum_{\text{i}=1}^{\text{n}}\;{\text{A}}_{\text{i}}-{\text{B}}_{\text{i}})/\text{n}=\text{error}$$

where data list A is the frequency counter output, data list B is the data transmitted to the base station PC, n is the data number used for comparison. Table 3 summarizes the mean error and standard deviation of the two data lists. Both the mean error and standard deviation between the transmitted data from the sensor node and the frequency counter output are less than 4 Hz.

## 4. Conclusions

We have reported a low-cost sensor node comprising a SAW sensor array, an interface chip, and wireless transmission module (Octopus II). The SAW sensor array comprised four SAW sensors with different sensing membranes operating non-continuously. The interface chip provided resolution in the frequency readout as low as 10 Hz. The wireless module transmitted sensor data to a remote computer for storage and analysis. This compact sensor node achieved high resolution, low power consumption, and is suitable for mass production and wireless sensor network applications.

## Figures and Tables

**Figure 2.**
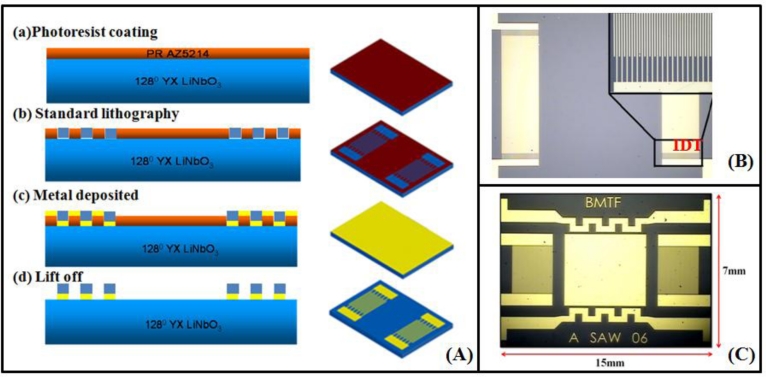
**(A)** The SAW device was fabricated using the standard photolithography process. **(B)** An enlarged optical image of interdigital transducers (IDTs). **(C)** Optical image of the SAW chip.

**Table 1.** Design parameters of the SAW chip. 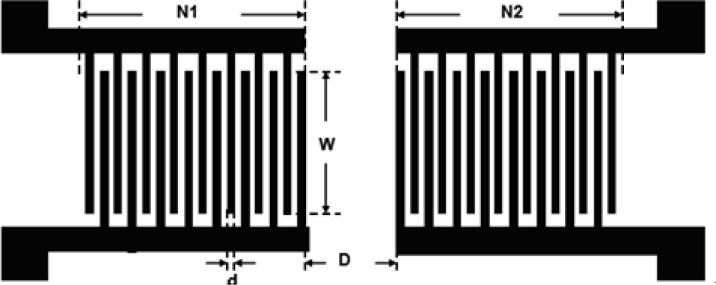

**Piezoelectric Substrate**	**C. F. (MHz)**	**λ(μm)**	**W(μm)**	**D(μm)**	**d(μm)**	**N1**	**N2**
						**Pair(s)**	
Au/LiNbO_3_(YX)	117.4	34	2074	4420	8.5	50	50

**Table 2.** Benchmark with other works of SAW interface circuit.

	[25]	[26]	This Work
Year	2005	2000	2010
Supply Voltage	3.3 V	2.5 V	3.3 V
Process Technology	0.35 μm	GaAs	0.18 μm
Power Consumption	38.35 mW	225 mW	1.48 mW
Resolution	10 MHz	3 MHz	10 Hz
Input Frequency	354 MHz	690 MHz	117.4 MHz

**Table 3.** Mean error and standard deviation of the two lists of data.

Gas	Ethanol		Acetone	
		
Readout data (Hz)	Mean Error	Standard deviation	Mean Error	Standard deviation

Membrane				

PNVP	2.18	2.56	2.41	2.47
PS	2.47	3.24	1.24	1.89
PSMA	2.48	3.39	2.17	2.52
PEG	2.13	3.25	1.25	1.44
P4VP	2.31	3.54	0.74	0.94
PVAc	1.53	2.69	1.23	1.85
PSu	1.97	2.58	3.68	2.94
